# Transcriptomics Reveals the Effect of Thymol on the Growth and Toxin Production of *Fusarium graminearum*

**DOI:** 10.3390/toxins14020142

**Published:** 2022-02-15

**Authors:** Lian-Qun Wang, Kun-Tan Wu, Ping Yang, Fang Hou, Shahid Ali Rajput, De-Sheng Qi, Shuai Wang

**Affiliations:** 1 Department of Animal Nutrition and Feed Science, College of Animal Science and Technology, Huazhong Agricultural University, Wuhan 430070, China; wlq2005199@126.com (L.-Q.W.); kuntanwu@webmail.hzau.edu.cn (K.-T.W.); yp923418@webmail.hzau.edu.cn (P.Y.); 2 Department of Animal Science, College of Animal Science and Technology, Tarim University, Aral 843300, China; liuyy221@126.com; 3 Faculty of Veterinary and Animal Science, Muhammad Nawaz Shareef University of Agriculture, Multan 60000, Punjab, Pakistan; shahid.ali@mnsuam.edu.pk

**Keywords:** thymol, *Fusarium graminearum*, deoxynivalenol, mycelial growth, toxin production, gluconeogenesis/glycolysis

## Abstract

*Fusarium graminearum* is a harmful pathogen causing head blight in cereals such as wheat and barley, and thymol has been proven to inhibit the growth of many pathogens. This study aims to explore the fungistatic effect of thymol on *F. graminearum* and its mechanism. Different concentrations of thymol were used to treat *F. graminearum*. The results showed that the EC_50_ concentration of thymol against *F. graminearum* was 40 μg/mL. Compared with the control group, 40 μg/mL of thymol reduced the production of Deoxynivalenol (DON) and 3-Ac-DON by 70.1% and 78.2%, respectively. Our results indicate that thymol can effectively inhibit the growth and toxin production of *F. graminearum* and cause an extensive transcriptome response. Transcriptome identified 16,727 non-redundant unigenes and 1653 unigenes that COG did not annotate. The correlation coefficients between samples were all >0.941. When FC was 2.0 times, a total of 3230 differential unigenes were identified, of which 1223 were up-regulated, and 2007 were down-regulated. Through the transcriptome, we confirmed that the expression of many genes involved in *F. graminearum* growth and synthesis of DON and other secondary metabolites were also changed. The gluconeogenesis/glycolysis pathway may be a potential and important way for thymol to affect the growth of *F. graminearum* hyphae and the production of DON simultaneously.

## 1. Introduction

Crops and food are often contaminated by molds and mycotoxins [1]. *Fusarium* head blight (FHB) is the most common disease of wheat in the world [2]. Some species of *Fusarium* can cause FHB, like *F. graminearum*, *F. culmorum*, and *F. avenaceum* [3]. The infection starts from the flowering period and progresses to the harvest period, causing the crops to be unharvested. A large amount of feed and food are rendered unfit for consumption due to mycotoxins contamination around the globe [4]. Finished feeds have high contamination rates and are often co-contaminated with multiple mycotoxins [5]. Common mycotoxins include aflatoxin B1 (AFB1) [6,7] and zearalenone (ZEN) [8], but deoxynivalenol (DON) and its acetyl-derivatives (3-Ac-DON and 15-Ac-DON) produced by *F. graminearum* are particularly concerning [9]. DON often contaminates wheat, barley, and oats. It can affect animal growth, immunity, and intestinal barrier function. In severe cases, it can induce vomiting, refusal to feed, and gastrointestinal bleeding in pigs [10]. Therefore, great attention is paid to controlling mycotoxins in feed to reduce economic losses. For compound feed for pigs, China’s Hygienical standard for feeds (GB 13078-2017) stipulates that the maximum limit is 1 mg/kg, and in the United States, it is 5 mg/kg. Due to the urgency of controlling FHB and the toxicity of trichothecenes, *F. graminearum* is listed as one of the top 10 fungal plant pathogens. Benzimidazole (such as carbendazim) and triazole (such as tebuconazole) have been widely used to control contamination by *F. graminearum* [11]. However, chemical fungicides are toxic and induce *F. graminearum* to synthesize DON [11]. Its residues on crops pose potential risks to environmental, animal, and human health. Therefore, people are paying more and more attention to finding biological antifungal agents alternative to synthetic pesticides. Researchers are particularly interested in essential oils (EO) extracted from plants, among natural fungistatic products. Due to its great potential to inhibit pathogens and their medicinal properties, it is considered one of the most promising biological antifungal agents.

EO is considered to be a secondary metabolite of aromatic flowers and plants. Because of their antiviral, fungistatic, and insecticidal properties in plants, they have been included in the category of natural preservatives [12]. Thymol (2-isopropyl-5-methylphenol) is a natural phenolic monoterpene compound, mainly found in *thymus Vulgaris*, *orange peel*, and *origanum* *heracleoticum* [13]. Thymol also has an excellent inhibitory effect on other toxin-producing molds, such as *Aspergillus flavus* [14], *Rhizoctonia solani*, *Pyricularia oryzae* [15], *Rhizopus stolonifera* [16], and *Fusarium solani* [17]. At the same time, it can also reduce the production of DON [18] and ZEN [19], so using thymol to inhibit *F. graminearum* has good potential and application prospects.

Some physical adsorbents can adsorb trichothecenes [20], but the effect is still not satisfactory. Adding thymol to feed can inhibit the mycelial growth of *F. graminearum* and reduce the production of trichothecenes from the source. It will be more conducive to the control of mycotoxins in the feed. Thymol is natural and degradable, and it also has the advantage of enhancing the antioxidant capacity of animals. Therefore, this experiment aims to study the effect of thymol on the growth and toxin production of *F. graminearum*. At present, there are also some reports that thymol inhibits the growth of *F. graminearum*, such as accumulating reactive oxygen species (ROS), destroying the integrity of cell walls and cell membranes [21], through ergosterol biosynthesis [22], and that it can block the overproduction of ROS [23]. However, there is a lack of research on other approaches. The fungistatic mechanism of EO is usually not a single pathway. Many studies have reported that EO can also change the membrane potential and destroy the integrity of cell membranes [24], inhibit DNA repair and transcription processes [25], or form chimeras with DNA and other pathways [26,27]. Therefore, we also adopted transcriptome sequencing technology (RNA-Seq), a high-throughput and high-resolution tool widely used to study fungi [28,29]. It can provide a comprehensive view of the *F. graminearum* transcriptome to comprehensively understand and explore other mechanisms by which thymol inhibits the growth of *F. graminearum*.

## 2. Results

### 2.1. The Effect of Thymol on the Growth of F. graminearum Hypha

**Figure 1:**
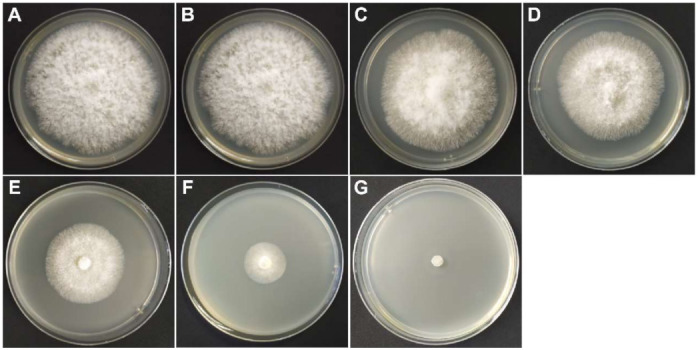
The effect of thymol on the growth of *F. graminearum* hyphae. (**A**) Control group; (**B**) 5 μg/mL thymol treatment group; (**C**) 10 μg/mL thymol treatment group; (**D**) 20 μg/mL thymol treatment group; (**E**) 40 μg/mL thymol treatment group; (**F**) 80 μg/mL thymol treatment group; (**G**) 160 μg/mL thymol treatment group.

**Figure 2:**
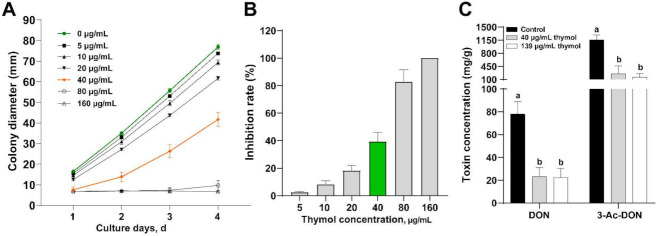
Thymol inhibits the growth of *F. graminearum* and DON synthesis. (**A**) Effect of different concentrations of thymol on the mycelial diameter; (**B**) Inhibition rate of different concentrations of thymol on the growth of *F. graminearum*; (**C**) The effect of thymol on the synthesis of DON and 3-Ac-DON at 40 μg/mL (EC_50_) or 139 μg/mL(EC_90_) concentrations. ^a,b^ Columns with different lowercase letters indicated significant differences between the compared groups (*p* < 0.05).

First, we added different concentrations of thymol to the medium to determine its inhibitory effect on *F. graminearum* (Figure 1). The results show that thymol had a good fungistatic effect, and the 10 μg/mL thymol treatment group significantly reduced the colony diameter on the fourth day (Figure 1C and Figure 2B). The inhibitory effect of thymol on the growth of *F. graminearum* had an obvious dose effect and time effect (Figure 1 and Figure 2A). At the same time, the inhibition rate of different concentrations of thymol on mycelial growth was calculated after the fourth day of culture (Figure 2B). The mycelial growth inhibition rate (MGIR) of *F. graminearum* reached 100%, and the thymol concentration was 160 μg/mL. The EC_50_ and EC_90_ calculated by the regression equation were 40.15 μg/mL and 139.12 μg/mL, respectively.

### 2.2. The Effect of Thymol on DON Production by F. graminearum

According to the previous growth inhibition test results, 40 μg/mL or 139 μg/mL of thymol was used to treat *F. graminearum* to evaluate its effect on DON. The results are shown in Figure 2C. The contents of DON and 3-Ac-DON in the control group were 78.0 ± 10.8 mg/g and 1160 ± 130.5 mg/g, respectively. The DON and 3-Ac-DON in the EC_50_ thymol treatment group were 23.3 ± 7.5 mg/g and 255.0 ± 209.3, respectively. Compared with the control group, the DON and 3-Ac-DON of the thymol treatment group decreased by 70.1% and 78.2%, respectively. The DON and 3-Ac-DON of the EC_90_ thymol treatment group were 22.3 ± 8.0 mg/g and 166.4.0 ± 91.6 mg/g, respectively, which decreased by 71.4% and 85.7%, respectively. The results show that 40 μg/mL thymol could significantly inhibit the production of DON and 3-Ac-DON by *F. graminearum*.

### 2.3. The Effect of Thymol on the Transcriptome of F. graminearum

To evaluate the quality of RNA-Seq data, we conducted some quality control analyses. Illumina sequencing produced 47,244,179 reads (control group) and 49,121,259 reads (thymol group). Strict data cleaning and quality inspection of the Illumina platform sequencing results, the error rate, GC percentage, and Q20 percentage were 0.02%, 52.2%, and 98.3%, respectively. Using Trinity to assemble all clean data de novo, a total of 16,727 non-redundant unigenes were identified, and the proportion of the expected length of the sequence to the total BUSCO score was 95.9%. The Mapped ratio between the sequencing data and the assembly results was 89.27%, indicating that this study’s assembled data were high quality.

**Figure 3:**
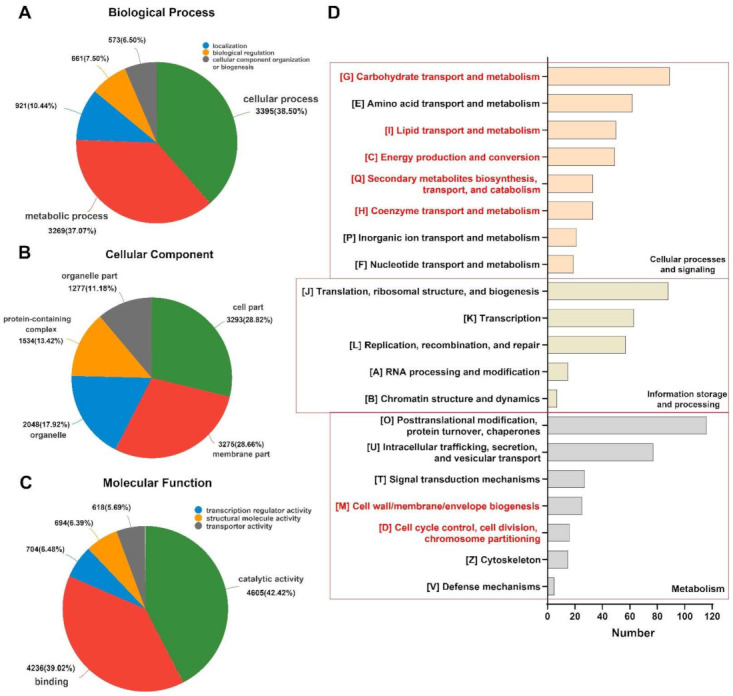
Function annotation analysis. (**A**–**C**) GO function annotation analysis; (**D**) COG function annotation analysis. Red markers represent annotations related to energy metabolism processes.

To better understand the functions of these non-redundant unigenes, all unigenes were compared with the NCBI non-redundant protein database sequences, and the e-value threshold was 10^−5^. The comparison analysis showed that a total of 12,597 unigenes matched the known proteins in the NR database. The matching percentages of *F. graminearum*, *Fusarium pseudograminearum*, and *Fusarium culmorum* were 73.05%, 5.61%, and 4.22%, respectively. All predicted unigenes were classified by functional annotation and classification through the Gene Ontology (GO) and Cluster of Orthologous Groups of Proteins (COG) database (Figure 3). The Top3 of the Biological Process (BP) were Cellular process, Metabolic process, and Localization; the Top3 of the Cellular Component (CC) were Cell part, Membrane part, and Organelle; the Top3 of the Molecular Function (MF) were Catalytic activity, Binding, and Transcription regulator activity. COG annotated 281, 230, and 356 unigenes with known functional classifications on Cellular processes and signaling, Information storage and processing, and Metabolism, respectively. In addition, 1653 (65.6%) unigenes were annotated by COG as Function unknown, and many unigenes were not matched to the database. These unigenes have the potential to be translated into functional proteins. This study’s RNA-seq data helps enrich the annotations of the unigenes group of *F. graminearum*. The qRT-PCR results of the candidate genes were compared with the corresponding RNA-seq data, and the results were the same (Figure S1 and Table S1), which confirmed th e accuracy of the expression profile based on the RNA-seq data.

**Figure 4:**
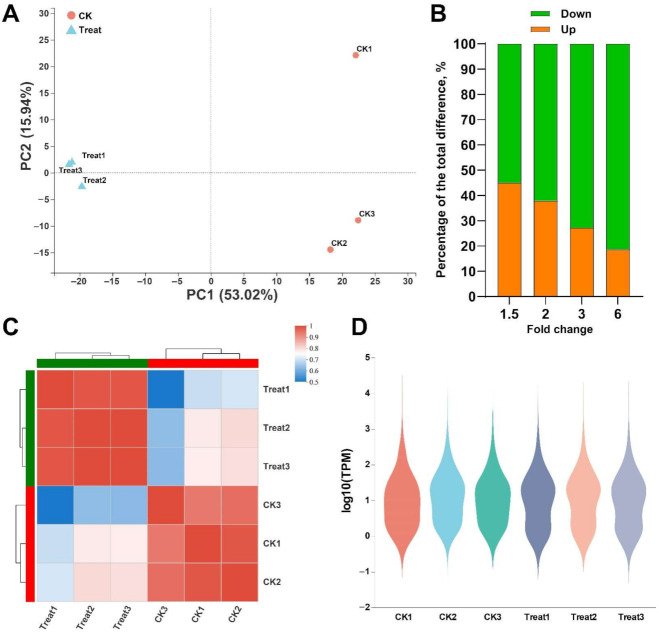
Analysis of the relationship between samples. (**A**) PCA analysis between samples; (**B**) Percentage of up-regulated/down-regulated unigenes identified by different multiples of difference; (**C**) Heat map of correlation analysis between samples; (**D**) Distribution of expression levels. CK is the control group, and Treat is the thymol treatment group.

RSEM quantitatively analyzed the expression level of the unigenes, and the quantitative index was TPM. The overall distribution diagram of the expression level is shown in Figure 4D. At the same time, the correlation of the expression levels between samples was analyzed, and the heat map results are shown in Figure 4C. The correlation coefficient between samples was >0.986 and >0.941 in the control and treatment groups, respectively. The results of the PCA are shown in Figure 4A. PC1 was 53.02%, and PC2 was 15.94%, indicating a high similarity between samples. These results suggest that the correlation between biological replicates was high and that the experimental design was reasonable. On this basis, the unigenes with a differential expression caused by thymol treatment were further screened out (Figure 4B), and *p*-adjust was <0.05. When Fold change (FC) = 1.5, a total of 4417 differential unigenes were identified, of which 1989 were up-regulated, and 2428 were down-regulated. When FC was 2.0 times, a total of 3230 differential unigenes were identified, of which 1223 were up-regulated, and 2007 were down-regulated. When FC was 3.0 times, a total of 1944 differential unigenes were identified, of which 529 were up-regulated and 1415 were down-regulated. When FC was 6.0 times, 884 differential unigenes were identified, of which 165 were up-regulated, and 719 were down-regulated.

**Figure 5:**
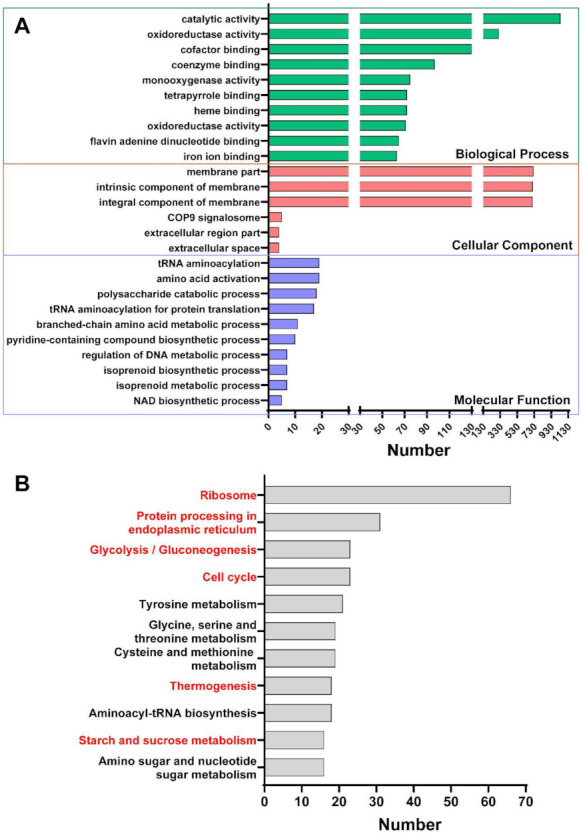
Functional enrichment analysis (**A**) GO function enrichment analysis; (**B**) KEGG function enrichment analysis.

First, we used a functional enrichment analysis to understand the differentially expressed unigenes’ functional pathways. The functional enrichment analysis of GO is shown in Figure 5A. The Top3 of BP were Catalytic activity, Oxidoreductase activity, and Cofactor binding. The Top3 of CC were Membrane part, Intrinsic component of membrane, and Integral component of membrane; the Top3 of MF were tRNA aminoacylation, Amino acid activation, and Polysaccharide catabolic process. The functional enrichment analysis of the Kyoto Encyclopedia of Genes and Genomes (KEGG) can be seen in Figure 5B. The Top3 of MF were Ribosome, Protein processing in endoplasmic reticulum, and Glycolysis/Gluconeogenesis.

### 2.4. Unigenes Related to Mycelial Growth and DON Production

The differentially expressed unigenes were functionally annotated and summarized in each NR, EggNOG, GO, KEGG, and Swiss-Prot database, and then the unigenes of interest were examined. Many unigenes related to mycelial growth (Table 1), DON synthesis (Table 2), secondary metabolites (Supplementary Materials Table S2), and glycolysis process (Supplementary Materials Table S3) were identified.

## 3. Discussion

### 3.1. The Effect of Thymol on the Growth of Mycelium

The fungistatic activity of thymol against pathogenic microorganisms has been widely reported [17,30]. For example, the EC_50_ of *Staphylococcus aureus*, *Staphylococcus luteus*, *Escherichia coli*, and *Bacillus cereus* is 27.64–128.58 μM [31]. Studies have found that seven kinds of thymol have suitable antifungal activities, one of which has an activity similar to the commercial fungicide thiabendazole [32]. Thymol inhibits the growth of *Fusarium oxysporum* with an EC_50_ of 80 µg/mL [21], and *Allium tuberosum R*. inhibits the growth of *Fusarium oxysporum* with an EC_50_ of 400 mg/mL [33]. In contrast, thymol has a better fungistatic effect. Thymol shows extensive antifungal activity against various isolates of *F. graminearum*, inhibiting the production of conidia, the growth of hyphae [20], and the production of DON [34]. By detecting the sensitivity of 59 *F. graminearum* strains to thymol, the EC_50_ values of thymol of these strains were 22.53–51.76 μg/mL, and the average value was 26.3 μg/mL [11]. In this study, the EC_50_ of thymol against *F. graminearum* was 40 μg/mL, compared with the EC_50_ of eugenol and its derivatives of 395.7–1163.9 μM, indicating that the sensitivity of *F. graminearum* to eugenol is lower than thymol [35]. Thymol can also be used in combination, such as cinnamaldehyde and carvacrol, as a synergist to enhance antifungal activity [36] and reduce the production of trichothecenes by 95–99% [24]. These studies have shown that thymol can very effectively inhibit the growth of *F. graminearum*, and it is a very potent plant and antifungal agent.

COG annotation results annotated many transcripts related to fungal growth, such as “Cell cycle control, Cell division, Chromosome partitioning” (16 unigenes) and “Cell wall/membrane/envelope biogenesis” (25 unigenes). From the Top10 of KEGG functional enrichment analysis, many transcripts were significantly enriched in “Protein processing in endoplasmic reticulum” (31 unigenes), and “Cell cycle” (23 unigenes) related to fungal growth was also screened (Figure 5B). Based on the functional annotations of related databases and previous research reports, we screened out unigenes associated with the effect of thymol on mycelial growth (Table 1). From the table, we can see that many related to cell growth processes, such as cycle control, the translation process, and ribosome and cell wall/membrane; cell division and replication processes, such as classification, chromosomes, and transcription processes; and material metabolism processes, such as carbohydrates, coenzymes, and lipids. The expression of unigenes during transportation and metabolism changed significantly.

Interestingly, COG annotated many unigenes related to “Ribosome” (66 unigenes). DON can interact with the peptidyl-transferase region of the 60 S ribosomal subunit to induce “ribosomal stress toxicity” [10]. It suggests that thymol may alleviate toxicity by alleviating the ribosomal stress caused by DON to animals [37]. However, the mechanism of action of thymol on the ribosomes of *F. graminearum* itself is still unclear, and further research is needed. The results show that thymol inhibits the growth of *F. graminearum* by affecting the expression of related unigenes in the various processes of mycelial growth.

### 3.2. The Effect of Thymol on DON Production by F. graminearum

During the process of *F. graminearum* infecting plants, it can produce a variety of secondary metabolites, and one of the most concerning products is DON [38]. Therefore, we also summarized the table where thymol affects mycelial DON (Table 2) and the synthesis of secondary metabolites (Supplementary Materials Table S2). *Tri* gene refers to a gene cluster related to the biosynthetic pathway of trichothecenes. *Tri1*, *Tri4*, *Tri13*, and *Tri11* are the more important CYPs in fungi. *Tri4* encodes a multifunctional oxygenase that converts trichodiene to isotrichotriol [39]. *Tri1* and *Tri11* encode 3-acetyltrich-othecen C-8 hydroxylase and isotrichodermin C-15 hydroxylase, respectively. *Tri13*, as the 3-acetyl trichothecenes C-4 hydroxylase, is responsible for the hydroxylation of C-4 [40]. *Tri4* is involved in the synthesis of the trichothecenes framework [34]. Compared with plants and animals, few fungal CYPs have been thoroughly studied for their functions. They may be the key enzymes fungi use to metabolize phenolic compounds and aromatic hydrocarbon compounds [41]. *Tri5* is the first gene in DON biosynthesis. *Tri6* and *Tri10* are unigenes that regulate the synthesis of DON [42]. Fusarium’s self-protection mechanism pumps the toxin out of the cell through *Tri12* and reduces the toxicity of intermediates in the biosynthesis of trichothecenes through *Tri101* [42]. Thymol can reduce the expression of *Tri5* [43] and inhibit the function of the toxin efflux pump, thereby enhancing the sensitivity of the fungus to tetracycline and benzalkonium chloride [44]. This is consistent with our results; 15 unigenes related to the fungal trichothecene efflux pump, such as *Tri10*, *Tri12*, *FUS6*, *FUB11*, and *ROQT,* were significantly down-regulated after thymol treatment. Studies have found that plant essential oils reduce the production of DON, 3-Ac-DON, and 15-Ac-DON by 96.33–100% [18], consistent with our results. The results indicate that thymol may inhibit the expression of unigenes clusters related to the trichothecene biosynthetic pathway and inhibit the Fungal trichothecene efflux pump, thereby inhibiting the synthesis of DON.

### 3.3. The Effect of Thymol on Glycolysis in F. graminearum

In addition to the synthesis of DON, thymol also affects the synthesis of many secondary metabolites [12]. For example, histone acetyltransferases, such as *Elp3*, *Sas3*, and *Gcn5*, are related to the regulatory effect induced by DON [45,46,47]. Earlier studies reported that thymol might cause cell membrane damage by inducing lipid peroxidation and inhibiting ergosterol biosynthesis, thereby inhibiting the growth of pathogenic fungus [22]. Interestingly, we found that thymol may also inhibit toxins’ growth and production by inhibiting the fungus’s glycolysis process through a further analysis of transcriptomics data. Supplementary Materials Table S3 found that the expression levels of many unigenes related to carbohydrates and protein methylases, acetylases, oxidoreductases, and hydrolases have undergone significant changes. *ADH* is responsible for catalyzing the last methanol synthesis step [48]. *ALDOC* participates in the aldol condensation reaction in glycolysis [49]. *NAGA* encodes α-N-acetylgalactosaminidase, which is mainly involved in regulating the metabolism of glycoproteins and glycolipids in lysosomes [50]. *PME* catalyzes the hydrolysis of pectin with pectinic acid and methanol. *DAK1* catalyzes the production of dihydroxyacetone phosphate and enters the glycolysis pathway. *ENOA* is the gene encoding enolase, the metallocenes responsible for catalyzing the ninth step of glycolysis, converting 2-phosphoglycerate to phosphoenolpyruvate. *CHI1* catalyzes the hydrolysis of chitin to N-acetylglucosamine. As a key enzyme in the glycolysis process, the phosphoglycerate kinase encoded by *PGK* can catalyze ATP production.

It shows that the synthesis of secondary metabolites is closely associated with gluconeogenesis/glycolysis. Cinnamaldehyde can regulate intracellular glucose metabolism through α-enolase [51]. *Chuzhou chrysanthemum* can inhibit the growth of *E. coli* through the hexose monophosphate pathway [12,52]. It proves that EO can hinder the growth of a fungus by affecting energy metabolism. Glycolysis is an important metabolic process of *F. graminearum*, so we infer that thymol should also be able to exert fungistatic effects through glycometabolism and energy utilization pathways (Supplementary Materials Table S3). The COG annotation results (Figure 3D) annotate that many unigenes are related to energy metabolism processes, such as “Coenzyme transport and metabolism” (33 unigenes), “Secondary metabolites biosynthesis, transport and catabolism” (33 unigenes), “Energy Production and conversion and Lipid transport and metabolism” (49 unigenes), “Amino acid transport and metabolism” (62 unigenes), and “Carbohydrate transport and metabolism” (89 unigenes). From the Top10 of the KEGG functional enrichment analysis, many unigenes were also screened to be significantly enriched in pathways related to the energy metabolism process (Figure 5B), such as “Starch and sucrose metabolism” (16 unigenes), “Thermogenesis” (18 unigenes), and “Glycolysis/Gluconeogenesis” (23 unigenes).

Studies have found that EO does not completely inhibit the production of AFB1 by inhibiting the growth of fungi. It may also interfere with the process of carbohydrate decomposition and metabolism, resulting in an insufficient supply of acetyl-CoA, thereby reducing the ability of fungi to produce aflatoxin [53]. This is because acetyl-CoA is a key component in the glycolysis process and a crucial substrate and raw material in the production of DON. Thymol can inhibit the expression of acetyl-CoA carboxylase and fatty acid synthase [54] and affect the utilization of farnesyl pyrophosphate FPP (the precursor of DON synthesis) [43]. Acetyl-CoA and Tri5 work together. Many studies have shown that thymol can affect many intermediate products in the tricarboxylic acid (TCA) cycle [17], such as citrate, fumarate, succinate, and α-ketoglutarate [21,55]. Thymol mediates its bactericidal activity against Staphylococcus aureus by targeting aldehyde-ketoreductase to consume NADPH [56]. On Caenorhabditis elegans, thymol accelerates glucose metabolism by regulating multiple targets in the glycolytic pathway and participates in the degradation of fatty acids [57]. In summary, thymol can affect the energy homeostasis in cells [58]. It may interfere with the glycolysis process and the formation of the DON toxin via acetyl-CoA or other common substances. This is a new idea to study the effect of EO on fungi. Acetyl-CoA is used as the raw material for DON synthesis. If the acetyl-CoA produced by glycolysis is not enough to supply its DON synthesis, it will eventually reduce the production of DON by *F. graminearum*. As far as we know, this is the first article combining thymol on the growth inhibition and toxin production of *F. graminearum* and RNA-Seq to understand the effect of thymol on *F. graminearum* fully.

## 4. Conclusions

Thymol can effectively inhibit the growth of *F. graminearum* and the production of DON. These results prove that after thymol treatment, many genes related to growth, DON, and the secondary metabolite synthesis process of *F. graminearum* undergo significant changes, which ultimately affect the growth and toxin production of *F. graminearum*. The study has enriched the data about thymol’s influence on the genes of *F. graminearum* from the transcriptomics level. In addition, since the acetyl-CoA produced by the gluconeogenesis/glycolysis process can simultaneously participate in growth and toxin production, we believe that gluconeogenesis/glycolysis can be a breakthrough point for future research on the regulation of other plant essential oils in *F. graminearum*.

## 5. Materials and Methods

### 5.1. Fungal Strain, Media and Culture Condition

The *F. graminearum* strain W3008 was kindly provided by the College of Plant Science and Technology of Huazhong Agricultural University, China [59]. The strain was routinely cultured at 25 °C on potato dextrose agar (PDA, Hopebio, Qingdao, China) plates and was preserved in 20% disinfected glycerol at −80 °C for long-term storage [60]. Thymol (HPLC grade standard, purity > 98%, B21153, Shanghai yuanye Bio-Technology Co., Ltd. Shanghai, China) was dissolved in acetone into a 100 mg/mL stock solution, protected from light, and stored at 4 °C.

### 5.2. Determination of the Sensitivity of Mycelial Growth to Thymol

According to the results of our previous experiments, thymol was diluted by a certain multiple and then added to the PDA medium. The control group only added an equal volume of acetone (0.5%); the final concentrations of the thymol treatment group were 0, 5, 10, 20, 40, 80, and 160 μg/mL, and the acetone concentration in all groups was 5 μL/mL (0.5% of acetone used). A 6 mm diameter bacterial cake was taken from the edge of a 3-day-old colony with a sterile puncher, placed in the culture medium’s center, and cultured for 4 days. The colony diameter was measured by the cross method every 24 h to evaluate the sensitivity of mycelial growth to thymol. The experiment was repeated 3 times, with 3 repetitions for each concentration. The percentage of mycelial radial growth inhibition on the 4th day of the culture after inoculation was calculated by the MGIR formula, MGIR (%) = [(C−N)/(C–6)] × 100. Where C and N are the average diameter values of the control and treatment groups, respectively. The thymol concentration of 50% (EC_50_) and 90% (EC_90_) of mycelial growth inhibition rate were calculated by the regression equation (See Supplementary Material “regression equation” and Figure S2).

### 5.3. Changes in DON and 3-Ac-DON

The preparation method of the conidia can be seen the supplementary material “Conidiation Assays” [46]. We added 1 mL of the spore suspension (5 × 10^5^ CFU/mL) to a flask containing 100 mL of GYEP (glucose yeast extract peptone) medium, and we incubated it with shaking (180 r/min) at 25 °C for 24 h [60]. Thymol was then added to the culture, and the same amount of acetone (0.5%) was added to the control culture. The final concentration of the thymol treatment group was 40 μg/mL (this concentration was close to the EC_50_) or 139 μg/mL (this concentration was close to the EC_90_). The acetone concentration in each culture was 5 μL/mL (0.5% of acetone used), and each treatment had 3 replicates. After 7 days of continuous cultivation, the mycelium was collected and dried at 60 °C for 3 h [61]. The filtrate was used for DON and 3-Ac-DON quantification. DON production in vitro was expressed as a ratio of DON content to dry mycelia weight (μg/g) [62]. The extraction and purification methods (see Supplementary Material “Changes in DON and 3-Ac-DON” for details) of DON and 3-Ac-DON were improved from Stroka [63], and the quantitative method was based on Diao [64]. The pure products of DON and 3-Ac-DON were from FERMENTEK, with a purity of >99.6%.

### 5.4. Transcriptome Analysis

A total of 100 mL of GYEP medium containing 1 mL of conidia suspension (5 × 10^5^ conidia/mL) was incubated with shaking (180 rpm/min) at 25 °C; after 24 h, thymol was added to the culture; the concentration of the thymol treatment group was 40 μg/mL, and the amount of acetone (0.5%) in groups control and treatment was same. Then, we continued incubating for 24 h, filtered, and collected hyphae [60]. The quality of RNA was evaluated after total RNA isolation using TRIzol reagent (Invitrogen, Shanghai, China). After the mRNA was isolated and fragmented, the library was sequenced using IlluminaHiSeq^TM^2000. Quality control of the original reading was performed, and we removed the linker and other low-quality base sequences to obtain the clean data reading.

Trinity was used to assemble all samples from scratch. TransRate and CD-HIT were optimized and filtered to remove common errors and redundancies. Then, BUSCO was used for assembly evaluation, and finally, the clean reads of each sample were compared with the reference sequence obtained by the Trinity assembly to obtain the mapping result of each sample. Unigene was the longest transcript in the transcript cluster, and unigenes were used for functional database annotation analyses (NR, Swiss-prot, Pfam, COG, GO, and KEGG). The RPKM method was used to calculate the expression value of unigenes (Reads Perkbper Millionread). RSEM compared the quality-controlled sequencing data with the assembled transcriptome sequence through comparison software, such as the bowtie, and then it estimated the expression abundance of unigenes/transcripts based on the comparison results. The quantitative expression index was TPM for homogenization so that the total expression in the sample was consistent for a more intuitive comparison. We used DESeq2 to analyze the differences between groups based on the quantitative expression results. The screening threshold was |log2FC| ≥ 1.585 and *p*-adjust < 0.05 to obtain unigenes with a differential expression between the two groups. Finally, the functional enrichment analysis of GO and KEGG was performed on the differentially expressed unigenes.

### 5.5. qRT-PCR

The total RNA was extracted from the sample as described above for real-time quantitative qRT-PCR analysis. Reverse transcription was performed using ABScriptIII Reverse Transcriptase kit (RK20408, ABclonal Technology Co., Ltd. Wuhan, China) with gDNA Eraser. Then, cDNA and 2×Universal SYBR Green Fast qPCR Mix reagent (RK21203, ABclonal Technology Co., Ltd.) were added to the 384 plates, respectively. We used a CFX384 real-time PCR system (Bio-Rad, Hercules, CA, USA) to complete qRT-PCR detection. The PCR program was as follows: 95 °C for 1 min, 40 cycles of 95 °C for 10 s, 60 °C for 5 s, and 72 °C for 10 s. The melting curve analysis was performed between 60 °C and 95 °C. The primers were synthesized by TSINGKE (Beijing, China). The qRT-PCR experiment was repeated 3 times, and each sample was repeated 3 times for analysis. *EF1α* was used as the reference gene for normalized expression data, and the relative gene expression level was calculated based on 2^−ΔΔCt^. Detailed information about gene-specific primers and alignment results are listed in the Supplementary Materials.

### 5.6. Statistical Analysis

The data were shown as mean ± standard deviation, comparing colony diameter, inhibition rate, DON, and 3-Ac-DON concentration. The values were analyzed by a one-way analysis of variance (ANOVA), followed by Duncan’s multiple range test, and different letters indicated significant differences at *p* < 0.05, compared with different concentrations of thymol. All analyses were performed with the GraphPad Prism 8.0 software (GraphPad Software, Inc., San Diego, CA, USA).

### 5.7. Availability of Supporting Data

The original Illumina sequencing dataset was submitted to the NCBI Sequence Read Archive with the accession number PRJNA792342. CK1-CK3 is the control group, and Treat1-Treat3 is the 40 μg/mL thymol treatment group.

## Figures and Tables

**Table 1:** Unigenes related to mycelial growth.

Name	COG_ID	FC	Swiss-Prot_Description	Functional Categories
CND2	COG5229	2.37	Condensin complex subunit 2	Chromatin structure and dynamics
MPIP	COG5105	3.05	M-phase inducer phosphatase	Cell cycle control, cell division, chromosome partitioning
HSK1	ENOG410YKCM	3.25	Cell cycle serine/threonine-protein kinase hsk1	
CDC45	ENOG410XR8E	2.00	Cell division control protein 45 homolog	
APC10	COG5156	3.50	Anaphase-promoting complex subunit 10	
CALM	COG5126	2.98	Calmodulin	
HXT2	ENOG410XNQK	0.46	High-affinity glucose transporter HXT2	Carbohydrate transport and metabolism
PHSG	COG0058	2.41	Glycogen phosphorylase	
HSP80	COG0326	0.06	Heat shock cognate protein 80	Coenzyme transport and metabolism
FSR4	ENOG4111F9D	2.44	Trans-enoyl reductase fsr4	Lipid transport and metabolism
PARP2	ENOG410XP18	13.54	ADP-ribose polymerase 2	Translation, ribosomal structure, and biogenesis
SMC2	COG1196	2.79	Structural maintenance of chromosomes protein 2	Transcription
SAK1	ENOG410XSHE	2.00	Protein sak1	
MCM1	COG5068	2.02	Transcription factor of morphogenesis MCM1	
ORC4	ENOG410XSK0	2.70	Origin recognition complex subunit 4	Replication, recombination, and repair
RAD1	ENOG410YHQU	2.26	DNA damage checkpoint control protein rad1	
SKP1	COG5201	2.42	E3 ubiquitin ligase complex SCF subunit scon-3	Posttranslational modification, protein turnover
CDK1	ENOG410XPP3	2.26	Cyclin-dependent kinase 1	Signal transduction mechanisms
PPZ	COG0639	0.49	Serine/threonine-protein phosphatase PP-Z	
PHLN	COG3511	0.08	Non-hemolytic phospholipase C	Cell wall/membrane/envelope biogenesis
GAL10	COG1087	3.03	Bifunctional protein GAL10	

**Table 2:** Unigenes related to the synthesis of DON.

Name	COG_ID	FC	Swiss-Prot_Description	Functional Categories
TRI4	COG2124	0.01	Cytochrome P450 monooxygenase	Cytochrome P450
TRI13	COG2124	0.02	Cytochrome P450 monooxygenase	
TRI11	COG2124	0.02	Trichothecene C-15 hydroxylase	
TRI1	COG2124	0.01	Cytochrome P450 monooxygenase	
TRI8	ENOG410YF7H	0.01	Trichothecene C-3 esterase	Trichothecene C-3 esterase
TRI6	ENOG410YRUA	0.01	Trichothecene biosynthesis transcription regulator 6	Trichothecene biosynthesis transcription regulator
TRI5	ENOG410YBAU	0.01	Trichodiene synthase	Trichodiene synthase
CLM1	ENOG410YEIS	0.01	Longiborneol synthase CLM1	
TRI3	ENOG4112AIP	0.01	Trichothecene 15-O-acetyltransferase	Trichothecene 15-O-acetyltransferase
TRI14	ENOG41118DK	0.03	Core trichothecene cluster	Core trichothecene cluster
TR101	ENOG410YJA8	0.09	Trichothecene 3-O-acetyltransferase	Transferase family
TRI10	ENOG410YFZP	0.06	Trichothecene biosynthesis transcription regulator 10	Fungal trichothecene efflux pump
TRI12	ENOG410XNQK	0.01	Trichothecene efflux pump TRI12	
ACLA	ENOG410XNQK	0.01	MFS efflux transporter aclA	
STR3	ENOG41109Q9	0.06	Siderophore iron transporter 3	
LACP	ENOG410XNQK	0.08	Lactose permease	
NAG3	COG0477	0.00	Major facilitator superfamily multidrug transporter NAG3	
GSFJ	ENOG410XNQK	0.08	Probable efflux pump gsfJ	
YJ94	ENOG410ZNSM	0.00	Uncharacterized membrane protein	
MF227	ENOG4111EZJ	0.02	Probable efflux pump mfs2	
ATB_A	COG0477	0.07	Efflux pump atB	
FUS6	ENOG410XNQK	0.06	Efflux pump FUS6	
FUB11	COG0477	0.06	Efflux pump FUBT	
MIRB	ENOG410XNQK	0.01	Siderophore iron transporter mirB	
ROQT	ENOG410XNQK	0.03	Efflux pump roqT	
SIT1	ENOG410ZYHG	0.15	Siderophore iron transporter 1	

## Data Availability

Not applicable.

## Supplementary Materials

The following supporting information can be downloaded at: https://www.mdpi.com/article/10.3390/toxins14020142/s1, Table S1: Primer sequence of quantitative fluorescence PCR; Table S2: Unigenes related to the synthesis of secondary metabolites; Table S3: Unigenes related to glycolysis; Figure S1: Comparison of gene expression (*Tri5*, *Tri6*, *Tri8*, *Tri14*, *Tri101*, *LEU1*, *6PGD1*, *ERG6*) levels based on RNA-seq and qRT-PCR; Figure S2: Sensitivity regression equation.
