# Supplementary material for: Transcriptomics Reveals the Effect of Thymol on the Growth and Toxin Production of Fusarium graminearum
Source: Toxins (Basel). 2022 Feb 15;14(2):142. doi: 10.3390/toxins14020142 (PMC8877954; doi:10.3390/toxins14020142)
Supplement: Supplementary file 1 [file toxins-14-00142-s001.zip › toxins-1552214-supplementary.pdf]

toxins

MDPI

Article

Transcriptomics Reveals the Effect of Thymol on the Growth and Toxin Production of *Fusarium graminearum*

Lian-Qun Wang, Kun-Tan Wu, Ping Yang, Fang Hou, Shahid-Ali Rajput, De-Sheng Qi and Shuai Wang

Table S1. Primer sequence of quantitative fluorescence PCR.

	Primer F (5'–3')	Primer R (5'–3')
EF1α	CTTCTTTCACCGCTCAGGTC	CTTGGAGGGAACCATCTTGA
Tri5	TTTTGGATGATAGCAGCGATG	CGAAGGACGTTGGGAAAGTGT
Tri6	GCTTATCGCCCTTCCCACC	ATGCCGCCTAAAGTCCCGT
Tri8	GATTTGAGAATGCCGAGCCT	TTGAAATGGCTGTCGGTCGT
Tri14	CGAACCTGCTGCTCTTACCG	TCGGTGGCGTGTCAGTTTAC
Tri101	GCACAACAACCCCGACAAGT	CGTAATCCCAGAGTCCCACC
LEU1	TACCAGTCAGAGGCTTCCGT	GTGGTGGTATGACTGTGGCA
6PGD1	CGAGTAAACACCTCCGCTATCT	GGTCAGTCCAACGCTGCAGT
ERG6	GAATGAGTTATCGGGAAAGGG	GTTGGATGTGGTGTTGGTGGA

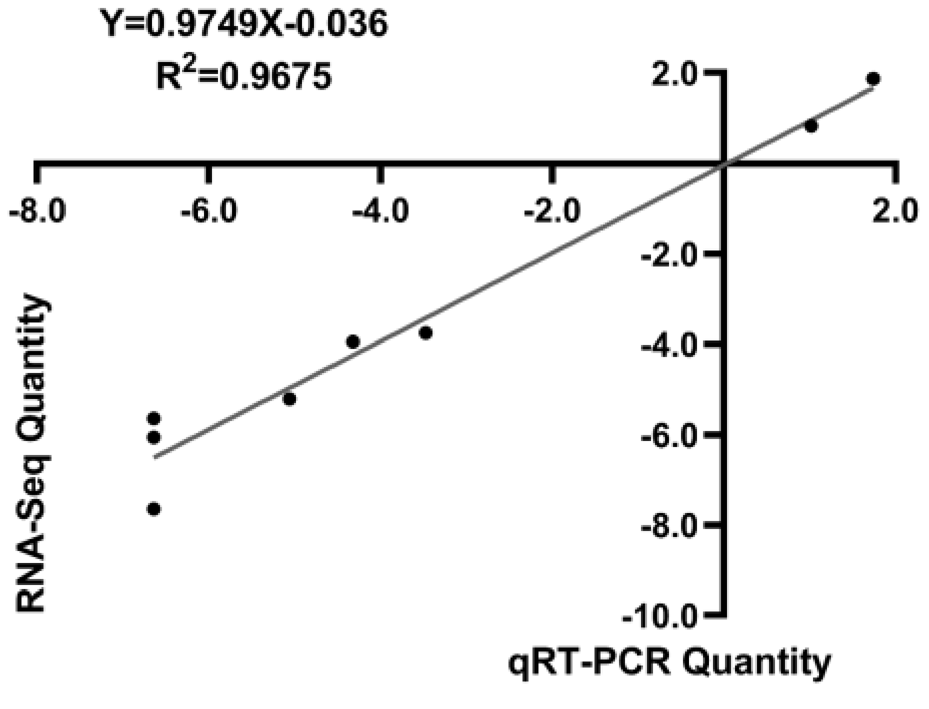

Figure S1. Comparison of gene expression (*Tri5*, *Tri6*, *Tri8*, *Tri14*, *Tri101*, *LEU1*, *6PGD1*, *ERG6*) levels based on RNA-seq and qRT-PCR.

Table S2. Unigenes related to the synthesis of secondary metabolites.

Name	COG_ID	FC	Swiss-Prot_Description	Functional Categories
ABCC	COG3207	0.62	ABC multidrug transporter C	ABC transporte
ALDC	COG3527	3.01	Alpha-acetolactate decarboxylase	α-acetolactate decarboxylase

FAC13	COG0318	0.33	Long-chain-fatty-acid--CoA ligase FadD13	AMP-binding enzyme
CLR3	COG0123	1.53	Histone deacetylase clr3	Arb2 domain;;Histone deacetylase domain
MPAS	COG3321	0.07	Methylphloroacetophenone synthase	Beta-ketoacyl synthase
AZAB	ENOG410XNPJ	2.40	Highly reducing polyketide synthase azaB	
AMO1	COG3733	1.67	Copper amine oxidase 1	Copper amine oxidase, enzyme domain
DLHH	COG0412	4.77	Putative carboxymethylenebutenolidase	Dienelactone hydrolase family
GSTK1	COG3917	0.30	Glutathione S-transferase kappa 1	DSBA-like thioredoxin domain
CRTI	COG1233	0.26	Phytoene desaturase	Flavin containing amine oxidoreductase
MNS2	ENOG410XP04	3.14	Mannosyl-oligosaccharide 1,2-alpha-mannosidase	Glycosyl hydrolase family 47
HOS3	COG0123	0.64	Histone deacetylase H	Histone deacetylase domain
TCMP	COG3315	0.17	Tetracenomycin polyketide synthesis O-methyltransferase TcmP	Leucine carboxyl methyltransferase
MET27	COG0500	0.18	Methyltransferase-like protein 27	Methyltransferase domain
ARYL	COG2162	16.00	Arylamine N-acetyltransferase, liver isozyme	N-acetyltransferase
PLCD1	ENOG410XPSW	1.61	1-phosphatidylinositol 4,5-bisphosphate phosphodiesterase delta-1	Phosphatidylinositol-specific phospholipase C
PLC1	ENOG410XPSW	0.45	1-phosphatidylinositol 4,5-bisphosphate phosphodiesterase 1	
FAD1	COG0175	2.25	Probable FAD synthase	Phosphoadenosine reductase family
CCD1	COG3670	0.08	Carotenoid 9,1	Retinal pigment epithelial membrane protein
XANA	COG2175	0.21	Alpha-ketoglutarate-dependent xanthine dioxygenase xan-1	Taurine catabolism dioxygenase TauD
URIC	COG3648	0.10	Uricase	Uricase

Table S3. Unigenes related to glycolysis.

Name	COG_ID	FC	Swiss-Prot_Description	Functional Categories
LEU1	COG0119	2.03	2-isopropylmalate synthase	Intracellular trafficking, secretion, and vesicular transport
DLD1	COG0277	2.31	D-lactate dehydrogenase, mitochondrial	Signal transduction mechanisms
PLC1	ENOG410XPSW	0.45	1-phosphatidylinositol 4,5-bisphosphate phosphodiesterase 1	Secondary metabolites biosynthesis, transport and catabolism
ADH	COG1064	0.30	Alcohol dehydrogenase	Posttranslational modification, protein turnover, chaperones
ALDOC	COG3588	4.76	Fructose-bisphosphate aldolase C	
CATA	COG0753	0.50	Peroxisomal catalase	Translation, ribosomal structure, and biogenesis
HIBCH	COG1024	2.08	3-hydroxyisobutyryl-CoA hydrolase, mitochondrial	Lipid transport and metabolism
DHCR7	ENOG410XP67	0.24	7-dehydrocholesterol reductase	

ACSA	COG0365	2.16	Acetyl-coenzyme A synthetase	
PHYT	COG4247	0.12	3-phytase	
AGALB	ENOG410XPF1	2.89	Probable alpha-galactosidase B	Carbohydrate transport and metabolism
HEX1	COG3525	0.43	Beta-hexosaminidase	
6PGD1	COG0362	3.35	6-phosphogluconate dehydrogenase, decarboxylating 1	
PME	COG4677	2.57	Pectinesterase	
INV2	COG1621	2.54	Beta-fructofuranosidase, insoluble isoenzyme 2	
BGLG_A	COG1472	2.97	Probable beta-glucosidase G	
CHI1	COG3325	0.08	Endochitinase 1	
TPSX	COG1877	0.35	Putative alpha,alpha-trehalose-phosphate synthase	
NAGA	COG1820	0.16	N-acetylglucosamine-6-phosphate deacetylase	
PHK	COG3957	0.28	Probable phosphoketolase	
DAK1	COG2376	3.47	Dihydroxyacetone kinase 1	
F16P	COG0158	2.14	Fructose-1,6-bisphosphatase	Nucleotide transport and metabolism
YLX7	COG1012	3.03	Putative aldehyde dehydrogenase-like protein	Energy production and conversion
ENOA	COG0148	3.51	Alpha-enolase	
PCKG	COG1274	2.68	Phosphoenolpyruvate carboxykinase	
FADH	COG1062	4.18	Zinc-binding dehydrogenase	
PGK	COG0126	5.90	Phosphoglycerate kinase	

Conidiation Assays

For conidiation Assays, ten mycelial plugs (6 mm diameter) taken from the periphery of a 3-day-old colony were transferred to a 250 mL flask containing 50 mL of carboxymethylcellulose sodium (CMC) liquid medium. The flasks were incubated in a shaker (180 rpm/min, 25 °C) for 7 days with the ambient laboratory light. After 7 days of cultivation, the liquid medium was filtered through three layers of sterile lens wiping paper, and the conidium was washed and resuspended with sterile water. The number of conidia was determined under microscopy by using a hemacytometer. The concentration of conidial suspension was adjusted to 5×10^5 conidia/mL in sterile distilled water for subsequent experiments of the DON production and the transcriptome of *Fusarium graminearum*.

Detection of DON and 3-Ac-DON

DON extraction and cleanup method, in brief, 5 g polyethylene glycol was added to the flask with a stopper containing 25 mL liquid medium, constant volume with water to 100 mL, the flask was placed in a shaker with 120 rpm/min for 30 min. After centrifuging at 3,000 rpm/min for 10 min, transfer 8 mL of the supernatant was passed slowly through an immunoaffinity column (M0608N3, Beijing Huaan Microbiotech Co., Ltd. , Beijing, China) immunoaffinity cleanup, and 4 mL of the purified extract was evaporated to dryness under a nitrogen stream. The dry residue was then redissolved in 1 mL of a methanol/water mixture (50:50, v/v) and filtered through a 0.22 μm nylon membrane filter. After that, it was ready for analysis.

DON quantification method, in short, the high-performance liquid chromatography (HPLC) system (Agilent 1200, Santa Clare, CA, USA) with a reverse-phase symmetry C_{18} column (5 μm, 150 × 4.6 mm, Agilent Eclipse XDB-C18) was applied to the quantitative analysis of DON and 3-Ac-DON. The column temperature was 30℃, the mobile phase

was water/methanol (82:18, v/v), and the flow rate was 0.7 mL/min with an injection volume of 20 μL. The detection wavelength was 218 nm, and the identification of DON or 3-Ac-DON was based on retention time decided by the peak time of the standard sample.

Regression equation

The thymol concentration of 50% (EC_{50}) and 90% (EC_{90}) of mycelial growth inhibition rate was calculated by the regression equation with Data Processing System (DPS, Hangzhou Reifeng Information Technology Ltd, Hangzhou, China). The regression equation and correlation coefficient are shown in Figure S2.

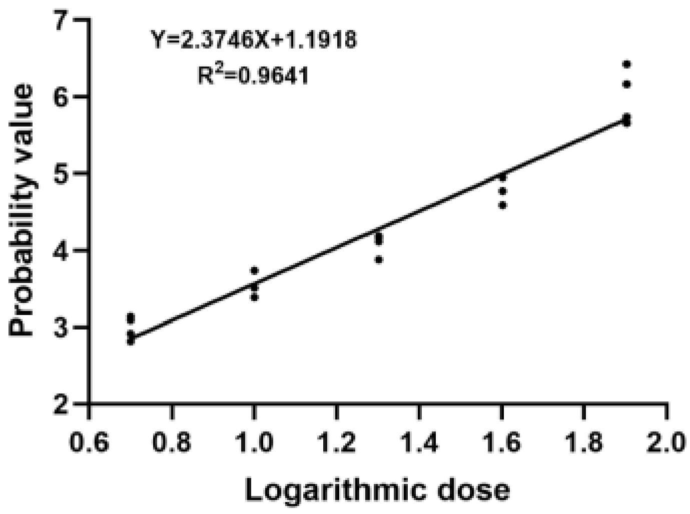

Figure S2. Sensitivity regression equation.
